# Childhood indicators of susceptibility to subsequent cervical cancer

**DOI:** 10.1038/sj.bjc.6600585

**Published:** 2002-10-21

**Authors:** S M Montgomery, A G C Ehlin, P Sparén, B Björkstén, A Ekbom

**Affiliations:** Enheten för klinisk epidemiologi, Institutionen för medicin vid Karolinska sjukhuset, Karolinska Institutet, Stockholm, Sweden; Institutionen för medicinsk epidemiology, Karolinska Institutet, Stockholm, Sweden; Centrum för allergiforskning & Institutet för miljömedicin, Karolinska Institutet, Stockholm, Sweden

**Keywords:** cervical cancer, HPV, warts, eczema, BCS70, NCDS

## Abstract

Common warts could indicate cervical cancer susceptibility, as both are caused by human papillomavirus (HPV). Eczema was also investigated, as atopic eczema has been negatively associated with warts, but non-atopic eczema may be associated with compromised host defences, as observed in patients with HIV, suggesting increased susceptibility to HPV infection and cervical cancer. ‘Cervical cancer’ was self-reported during an interview by 87 of 7594 women members of two longitudinal British birth cohorts. The accuracy of the diagnoses is limited by lack of confirmation using medical records. Odds ratios are adjusted for common warts and eczema in childhood; and cigarette smoking, number of cohabiting partners and social class in early adult life. The odds ratios of warts and eczema with cervical cancer are 2.50 (95% confidence interval 1.14–5.47) and 3.27 (1.95–5.49), respectively. The association of eczema with cervical cancer is independent of hay fever as a marker of atopy, suggesting the importance of non-atopic eczema. Both heavier smoking compared with non-smoking and four or more cohabiting partners compared with one/none have odds ratios for cervical cancer of 8.26 (4.25–15.10) and 4.89 (1.39–17.18), respectively. Common warts in childhood may indicate cervical cancer susceptibility; this and the relationship with eczema deserves investigation.

*British Journal of Cancer* (2002) **87**, 989–993. doi:10.1038/sj.bjc.6600585 www.bjcancer.com

© 2002 Cancer Research UK

## 

This study sought to identify childhood susceptibility to subsequent cervical cancer using putative markers for susceptibility to persistent viral infections, as cancer of the cervix is causally linked with Human papillomavirus (HPV) (Bosch *et al*, 1995). A diagnosis of common warts was chosen as a marker of susceptibility as warts are caused by HPV infection (Chan *et al*, 1994) and genital warts are also associated with an increased risk of cervical cancer (Franceschi *et al*, 1983; Kjaer *et al*, 1992).

Childhood eczema was also investigated as a potential marker of immune function relevant to susceptibility to HPV infection, the *a priori* hypothesis being that it was prospective, any association with cervical cancer might vary by eczema type. We hypothesised that atopy, particularly atopic eczema, might confer protection against cervical cancer as one study reported an inverse association between atopic eczema and common warts, (Williams *et al*, 1993) suggesting atopy may protect against HPV infection. In contrast to a suggested protective role of atopic eczema, non-atopic types such as seborrhoeic eczema may indicate compromised host defences and represent an increased risk for persistent HPV infection and cervical cancer. Some eczema types are associated with susceptibility to herpes simplex virus infection and eczematous lesions are common in patients with compromised host defence (Dahl, 1996; Uthayakumar *et al*, 1997; Shimizu *et al*, 2000) or HPV-2 infection (Rubben *et al*, 1997). We used a diagnosis of hay fever as a marker of atopy to assist in the interpretation of any association between eczema and cervical cancer to determine if atopy was a confounding factor or effect modifier.

The study used two general-population birth cohorts to investigate associations of warts and eczema in childhood with subsequent cervical cancer. Data on putative childhood susceptibility markers were collected prospectively in childhood, prior to any form of cervical cancer. The diagnosis of ‘cervical cancer’ was subsequently self-reported by these women in adulthood. Data on warts were available in one cohort and on eczema in both.

## MATERIALS AND METHODS

The data are from two British longitudinal birth cohort studies: the National Child Development Study (NCDS) and the 1970 British Cohort Study (BCS70), which follow everyone in Great Britain born in the weeks 3–9 April 1958 and 5–11 March 1970, respectively (Ekinsmyth *et al*, 1992; Ferri, 1993; Bynner *et al*, 1998). Each study is based on approximately 16 000 individuals followed from birth to 42 or 30 years in NCDS and BCS70, respectively (Ekinsmyth *et al*, 1992; Ferri, 1993; Bynner *et al*, 1998). After exclusion of males and those with incomplete data, some 3654 and 3941 were available for analysis in NCDS and BCS70, respectively. Response rates varied by sweep and have been reported in detail elsewhere (Ekinsmyth *et al*, 1992; Ferri, 1993; Bynner *et al*, 1998). Totals of 11 419 (5795 female) and 11 261 (5790 female) cohort members participated in data collection sweep at ages 42/30 years in NCDS and BCS70, respectively. Despite greater loss from more disadvantaged groups, the cohorts are largely representative (Ferri, 1993; Bynner *et al*, 1998). However, in the data used here, the proportion of females from a family in social class V (the most disadvantaged) at birth declined from 8.5 to 7.3% and 5.0 to 4.0% in NCDS and BCS70, respectively.

In summary, the following data were used (and collected at these ages). NCDS: eczema and common warts (11 and 16 years); diagnosis of any cancer, hay fever and smoking (16 years); social class and cohabiting partners (33 years); and cervical cancer after age 17 years (42 years). BCS70: eczema (5 and 10 years); diagnosis of any cancer and hay fever (10 years); smoking, social class, cohabiting partners and cervical cancer after age 11 years (30 years).

A diagnosis of ‘cervical cancer’ was reported during interviews at ages 42 years in NCDS and 30 years in BCS70. Eczema and common warts (on arms and legs) were identified in NCDS by medical examination conducted by a local authority medical officer at 11 and 16 years. As warts were assessed by a medical examination at two time points, rather than record review, the estimates of prevalence for warts may be conservative, but a positive record is likely to be accurate. Hay fever was recorded at age 16 years in NCDS. In BCS70, eczema at age 5 years was identified by health visitor interviews with parents and medical records. At age 10 years, community medical officers and school nurses recorded a diagnosis of eczema during a medical examination. Hay fever was recorded at age 10 years in BCS70. A history of chronic disease, including cancer, was recorded from review of medical records at ages 16 years and 10 years in NCDS and BCS70, respectively.

Cigarette smoking was reported in NCDS at age 16 years by a self-completed questionnaire. Number of cigarettes smoked was dichotomised into under three or three and more packets of 20 per week. In BCS70, smoking history was recorded by interview at age 30 years and dichotomised into less than a packet of 20 or at least a packet of 20 cigarettes smoked per day. The number of cohabiting partners to date was recorded during interviews at ages 33 and 30 years for NCDS and BCS70, respectively. Social class using the Registrar General's classification was based on current or most recent job at ages 33 and 30 years for NCDS and BCS70, respectively. The social class measure was divided into three groups: non-manual, manual and class not ascertained.

### Statistical analysis

Women with cancer of any type by age 16 years in NCDS or 10 years in BCS70 were excluded (one woman in BCS70). Cervical cancer was the dependent variable in multiple logistic regression using SPSS (Norusis, 1989). Adjustment was made for the susceptibility measures and potential confounding factors, modelled as series of binary dummy variables. Additional analysis adjusted for hay fever.

A combined analysis of both cohorts required modification of some variables for comparability. As common warts were only assessed in NCDS, the wart status for all those in BCS70 was set as not known. This also introduced an adjustment for cohort and thus age at follow-up. Cigarette smoking was recoded so that never-smokers were in the no smoking category; ex-smokers, occasional smokers and those smoking less than three packets per week at 16 years or less than one packet per day at 30 years were classified as moderate smokers; the other smokers were combined in a heavy smoker category. In examining the association of single factors with cervical cancer without adjustment for potential confounding, adjustment was made for cohort (and thus age) using a dummy variable for cohort.

## RESULTS

### NCDS

Table 1

**Table 1:**
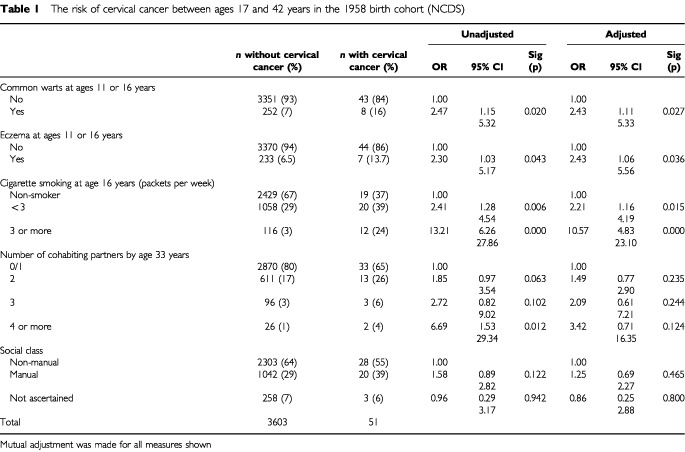
The risk of cervical cancer between ages 17 and 42 years in the 1958 birth cohort (NCDS)

shows odds ratios for ‘cervical cancer’ from age 17 to 42 years. Common warts and eczema at ages 11 or 16 years were statistically significantly associated with cervical cancer. If common warts and eczema are combined into a single variable, those with either common warts or eczema have an adjusted odds ratio for cervical cancer of 2.50 (1.21–5.14) and those with both common warts and eczema have an adjusted odds ratio of 10.21 (1.22–85.54).

There is a graded relationship between numbers of cigarettes smoked per week at age 16 years and cervical cancer risk. There is a graded and weakly statistically significant relationship with number of cohabiting partners. Statistical significance for partners is reduced in the adjusted model, but the trend across categories remains statistically significant with an increase in odds of 1.48 (1.01–2.16). There is a positive association of smoking with number of partners cohabitating (*P*<0.001) and the co-linearity of these factors reduces their odds with adjustment. There is no statistically significant relationship of cervical cancer with social class.

Some 358 women had hay fever by age 16 years, which was not significantly associated with cervical cancer (*P*=0.793). Additional adjustment for hay fever increases the odds ratio for eczema with cervical cancer to 2.54 (1.02–6.32).

### BCS70

Table 2

**Table 2:**
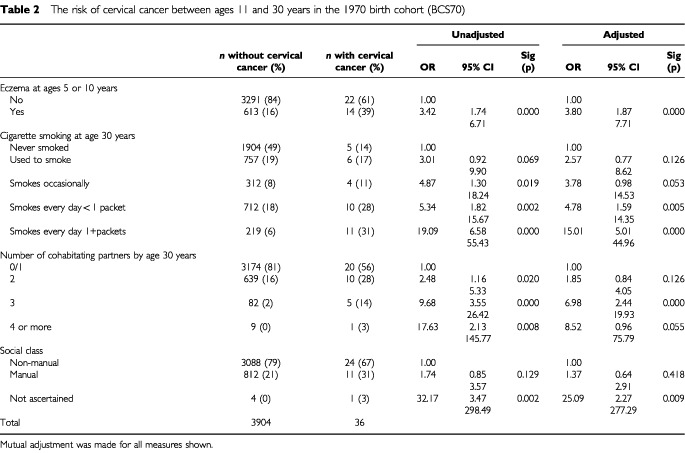
The risk of cervical cancer between ages 11 and 30 years in the 1970 birth cohort (BCS70)

shows odds ratios for ‘cervical cancer’ after age 11 and by 30 years. Eczema at 5 or 10 years of age is statistically significantly associated with subsequent cervical cancer.

Women who were smoking at least one packet of 20 cigarettes per day at age 30 years were substantially more likely to have a self-reported diagnosis of ‘cervical cancer’. Again, the positive relationship between number of cohabiting partners and cervical cancer risk is diminished by adjustment for smoking as these factors are collinear, but the relationship of number of partners and cervical cancer remains statistically significant after adjustment.

Some 305 had hay fever by age 10 years, which was not associated with self-reported ‘cervical cancer’ (*P*=0.807). Adjustment for hay fever increases the odds ratio for eczema with cervical cancer to 3.93 (1.91–8.10).

### Combined analysis

As common warts were not assessed in BCS70, the estimates for their association with cervical cancer are unaltered by combining the two cohorts. The precision of the estimates for eczema is improved and indicates a robust positive relationship with subsequent cervical cancer (Table 3

**Table 3:**
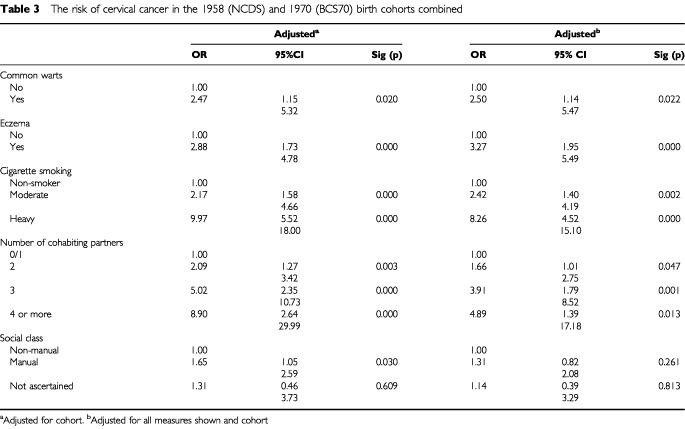
The risk of cervical cancer in the 1958 (NCDS) and 1970 (BCS70) birth cohorts combined

).

The collapsing of categories necessary to combine the cohorts, accounts for the reduction in odds ratios for cervical cancer in some smoking groups, although this positive association remains highly statistically significant. Number of cohabiting partners remains significantly positively associated with cervical cancer after adjustment for the potential confounding factors. A slight reduction in the impact of adjusting for smoking on the association between number of cohabiting partners and cervical cancer is due to the collapsed smoking categories.

The increased statistical power of the combined model reveals a statistically significant association between manual social class and cervical cancer, but this was eliminated by adjustment for the potential confounding factors.

Hay fever was not significantly associated with cervical cancer in the combined analysis, with an odds ratio of 1.04 (0.50–2.17), *P*=0.922 and is not a confounding factor.

## DISCUSSION

This study found that common warts in childhood were associated with subsequent cervical cancer. As human papillomavirus (HPV) is causally linked with cervical cancer (Bosch *et al*, 1995) we hypothesised that common warts in childhood may indicate vulnerability to this group of viruses and thus cervical cancer, as common warts are also caused by HPV infection. (Chan *et al*, 1994) Genital warts are a known risk for cervical cancer, (Franceschi *et al*, 1983; Kjaer *et al*, 1992) but we believe that this is the first report of an association with common warts. Unlike genital warts associations with common warts in childhood are not confounded by sexual activity and related lifestyle risks.

A positive association between childhood eczema and subsequent ‘cervical cancer’ was also observed. As this association was observed in both cohorts, it is unlikely to be a chance finding, though an initial hypothesis was that it was protective. It is likely that the type of eczema associated with cervical cancer is not atopic as adjustment for hay fever did not diminish the association with cervical cancer and hay fever was not associated with cervical cancer. Further evidence suggesting that this is not atopic eczema is a previously reported negative association between common warts and atopic eczema in the 1958 cohort (Williams *et al*, 1993) although there is some evidence to suggest that the relationship of common warts with allergy and eczema may be influenced by HPV strain (Rubben *et al*, 1997). Studies of the relationship between atopy and cancer have reported inconsistent results, (Källén *et al*, 1993; Mills *et al*, 1992; Vesterinen *et al*, 1993; Eriksson *et al*, 1995) but the risk may vary by allergy type (Mills *et al*, 1992) and none of the studies of atopy and cancer cited here specifically investigated eczema. In some studies, allergy was too loosely defined, as the ‘allergies’ do not have a common mechanism. Because hay fever is associated with atopy in children and adolescents, we used this as a more reliable indicator of atopy. The lack of association with hay fever suggests that the eczema predictive of subsequent cervical cancer occurs both in atopic and non-atopic women. This may be seborrhoeic eczema, which is possibly due to an inability to control yeast colonisation and individuals with this type of eczema may have depressed T-cell function (Bergbrant *et al*, 1991).

Non-atopic eczema in childhood could be a marker of vulnerability for cervical cancer as some eczema types may indicate compromised host defence. Seborrhoeic eczema is more common in patients with HIV (Dahl, 1996; Uthayakumar *et al*, 1997; Shimizu *et al*, 2000) indicating that this type of eczema may reflect susceptibility to persistent infection as in HIV patients with impaired immune function. Here, it is often described as atypical and seborrhoeic disease (Uthayakumar *et al*, 1997). Eczema has also been associated with HPV-2 infection (Rubben *et al*, 1997).

This study benefits from the use of two general population-based cohorts of individuals followed from birth to adult life, representing a rare source of prospectively collected childhood data that can be linked with adult disease outcomes. A disadvantage is that cervical cancer was self-reported by women and was not medically confirmed. Thus, there is no information on the histological type of cervical cancer, nor whether it is invasive or, as often found in these age groups, *in situ*. However, HPV infection is implicated in the aetiology of these types, although there may be some differences in HPV strain (Thomas *et al*, 2001). The prevalence of ‘cervical cancer’ in this study is almost certainly inflated by cervical intraepithelial neoplasias (CIN) or other milder types of dysplasia, which women had not distinguished from (invasive) cancer of the cervix. It is unlikely that attrition of the cohort has increased substantially the proportion with cervical cancer as the relatively socially disadvantaged are more likely both to be lost to follow up (Ferri, 1993; Bynner *et al*, 1998) and develop cervical cancer (Krieger *et al*, 1999).

To take into account the possible influence of recognised risks for cervical cancer, we adjusted our analyses for measures of cigarette smoking (Deacon *et al*, 2000) and adult social class. As greater numbers of sexual partners increase the risk of HPV infection (Deacon *et al*, 2000), we also adjusted our analyses for number of cohabiting partners in adult life as an indicator of number of sexual partners. By combining the cohorts we were able to increase statistical power and improve the accuracy of some estimates. A statistically significant increased risk of cervical cancer among women with a lower social class was only apparent and well estimated in the combined analysis. The statistical significance was eliminated by adjustment for the potential confounding factors indicating that the components of social class associated with cigarette smoking and sexual activity are important in explaining social class gradients in cervical cancer risk.

Data on cigarette smoking were collected prior to a diagnosis of cervical cancer in NCDS and at a later age in BCS70, although the associations with cervical cancer were similar. NCDS women smoking three or more packets of cigarettes per week at age 16 years have a substantially increased risk of subsequent cervical cancer. Despite the reduction in precision necessary for combined analysis of the smoking data, the greater statistical power on the combined analysis provides evidence that both cigarette smoking and having multiple sexual partners are independent risks for cervical cancer. The co-linearity of smoking with number of cohabiting partners confirms that smoking is also a marker for sexual activity, such that women who smoke more cigarettes also have a greater number of sexual partners.

HLA associations for ‘cervical cancer’ suggest that class II genes are important in determining whether HPV infections are cleared, probably through antigen presentation to helper T cells (Breitburd *et al*, 1996); defects in antigen presentation might result in T-cell anergy (Nickoloff and Turka, 1993, 1994) increasing the risk of persistent HPV infection. It is plausible that this is reflected in a greater susceptibility to common warts. The association of childhood eczema with cervical cancer may also indicate greater susceptibility to persistent HPV infection. A plausible mechanism is less obvious here, but individuals with compromised host defence may manifest some types of eczema: further research is required to confirm this association with an emphasis placed on identifying the relevant eczema type. With refinement, one or both of the markers suggested by this study may provide simple means to quantify susceptibility to cervical cancer without HLA typing.
